# *In-silico *and *in-vivo *analyses of EST databases unveil conserved miRNAs from *Carthamus tinctorius *and *Cynara cardunculus*

**DOI:** 10.1186/1471-2105-13-S4-S12

**Published:** 2012-03-28

**Authors:** Domenico Catalano, Domenico Pignone, Gabriella Sonnante, Mariella M Finetti-Sialer

**Affiliations:** 1 Istituto di Genetica Vegetale, Consiglio Nazionale delle Ricerche, Bari, 70126, Italy

## Abstract

**Background:**

MicroRNAs (miRNAs) are small RNAs (21-24 bp) providing an RNA-based system of gene regulation highly conserved in plants and animals. In plants, miRNAs control mRNA degradation or restrain translation, affecting development and responses to stresses. Plant miRNAs show imperfect but extensive complementarity to mRNA targets, making their computational prediction possible, useful when data mining is applied on different species. In this study we used a comparative approach to identify both miRNAs and their targets, in artichoke and safflower.

**Results:**

Two complete expressed sequence tags (ESTs) datasets from artichoke (3.6·10^4 ^entries) and safflower (4.2·10^4^), were analysed with a bioinformatic pipeline and *in vitro *experiments, identifying 17 potential miRNAs. For each EST, using RNAhybrid program and 953 non redundant miRNA mature sequences, available in mirBase as reference, we searched matching putative targets. 8730 out of 42011 ESTs from safflower and 7145 of 36323 ESTs from artichoke showed at least one predicted miRNA target. BLAST analysis showed that 75% of all ESTs shared at least a common homologous region (E-value < 10^-4^) and about 50% of these displayed 400 bp or longer aligned sequences as conserved homologous/orthologous (COS) regions. 960 and 890 ESTs of safflower and artichoke organized in COS shared 79 different miRNA targets, considered functionally conserved, and statistically significant when compared with random sequences (signal to noise ratio > 2 and specificity ≥ 0.85). Four highly significant miRNAs selected from *in silico *data were experimentally validated in globe artichoke leaves.

**Conclusions:**

Mature miRNAs and targets were predicted within EST sequences of safflower and artichoke. Most of the miRNA targets appeared highly/moderately conserved, highlighting an important and conserved function. In this study we introduce a stringent parameter for the comparative sequence analysis, represented by the identification of the same target in the COS region. After statistical analysis 79 targets, found on the COS regions and belonging to 60 miRNA families, have a signal to noise ratio > 2, with ≥ 0.85 specificity. The putative miRNAs identified belong to 55 dicotyledon plants and to 24 families only in monocotyledon.

## Background

The family *Asteraceae *represents one of the largest evolutive radiations of flowering plants, including more than 1.5·10^3 ^genera and 2.3·10^4 ^species, comprising economically important as well as ornamental crops [1,2]. Among members of this dicotyledon family two crop species belonging to the Cardueae tribe, *Carthamus tinctorius *L. and *Cynara cardunculus *var. *scolymus *L., also hold a phyto-pharmaceutical interest. The former, known as safflower, is the only member of this genus widely cultivated for industrial oil, as a livestock feed or for use in traditional medicine [3]. The second species, the globe artichoke, apart from its importance as food, is popular for its dietary and therapeutic potentials, especially for hepato-biliary dysfunctions and digestive complaints [4,5].

Significant progress has been achieved in recent years on the mechanisms of gene regulation and expression in plant and animals [6,7] and several studies already elucidated gene regulation in the model organisms *Caenorhabditis elegans *and *Arabidopsis thaliana *[8,9]. Few data are available thus far on gene expression [10] or regulation based on small regulatory elements, on a wide scale, for *C. cardunculus *and *C. tinctorius*.

A class of tiny RNA molecules, *lin-4 *and *let-7*, both controlling the timing of juvenile development in *C. elegans *were the founding members of non-coding RNAs called microRNAs (miRNAs) [11-13]. Plant miRNAs came to light from *A. thaliana *studies [13]. Their discovery broadened the phylogenetic distribution of miRNAs to plant genomes and highlighted their ancient origin and the important role played [14]. Recently, a significant advancement was achieved through the discovery that small RNA molecules were not only active within the cell, but also as mediators at the cell-to-cell communication level [15]. Plant miRNAs derive from long primary transcripts (pri-miRNA) giving rise to mature RNAs products of 21-24 bp, fundamental in gene regulation [16]. In plants, miRNAs control the degradation of messengers or restrain translation, affecting development and response to biotic and abiotic stresses [17].

The biogenesis of miRNAs in plant is a multi step process: initially a miRNA gene is transcribed in the nucleus (pri-miRNA) by means of POLII enzyme [16,18]. Subsequently, the pri-miRNA is cleaved in the miRNA precursor or "pre-miRNA". In this step the precursor is organized in a typical stem loop structure. A Dicer-like enzyme is involved in the plant miRNA maturation, functioning in concert with the dsRNA binding protein (dsRBP) [18,19]. After this step, the processed miRNA is transported to the cytoplasm by the HASTY protein [20] to build a complex, termed "RNA-induced silencing complex (RISC)", with ARGONAUTE (AGO) proteins. The RISC complex guide has a functional activity of cleavage or translational repression of its cognate mRNA target by base-paring. In plants, miRNAs have several roles involved in most biologic processes [21] as well as in diverse signalling networks, like leaf [22,23] and flower development [24,25].

**Figure 1:**
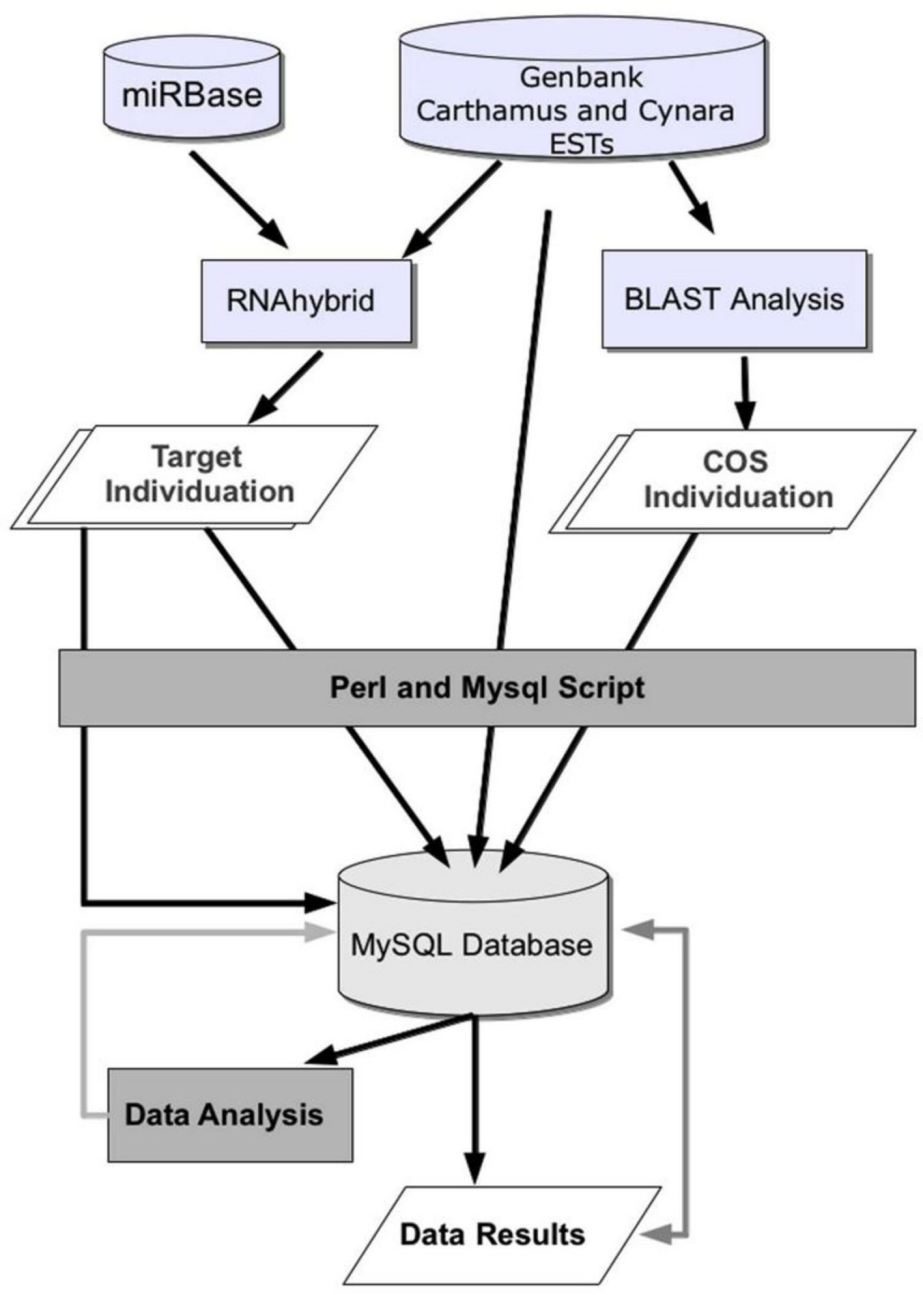
**Workflow of the computational approach followed to identify miRNAs**. Overall procedure for miRNA, target and homologous regions identification.

Unlike animal mRNA targets, plant targets show a single sequence motif displaying a near-perfect complementarity to their miRNAs. The imperfect but extensive correspondence of plant miRNAs to their mRNA targets provides a feature which makes a computational prediction possible [26,27]. This approach is useful when data mining is performed on the basis of miRNA:mRNA targets conservation among different species, and was herein applied to the study of globe artichoke and safflower. In this study, miRNA identification and analysis were developed through a primary tool, RNAhybrid [28], in order to find all available targets. In a subsequent step we looked for homologous Expressed Sequence Tags (ESTs), in both species. Finally, by means of a bioinformatics pipeline and a relational database, the presence of single targets in homologous ESTs regions was matched, in both species. Finally, some of the identified sequences were validated by means of experimental assays. The overall procedure for miRNA, target and homologous regions identification is explained in Figure 1.

## Results and discussion

### *In silico *miRNAs identification

**Figure 2:**
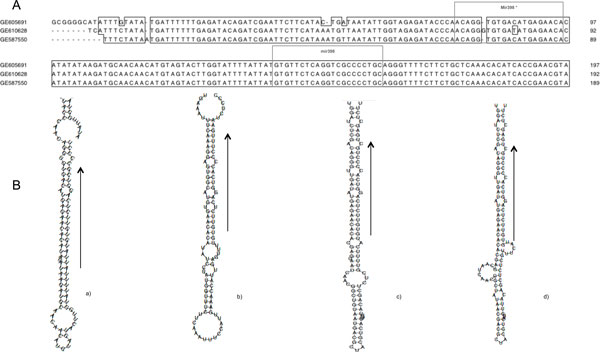
**EST alignment of selected sequences of *Cynara cardunculus *and comparison with *Arabidopsis *stem-loop structures**. **A**. Partial sequence alignment of precursors from *Cynara cardunculus *corresponding to stem-loop of miR398. **B**. Stem-loop structures for miR398 (mfe -48.57 kcal/mol) (a), and Ath-miR398a,b,c (mfe -39.84, -51.50 and -46.10 kcal/mol, respectively) (b-d). Arrows mark mature miRNA sequences.

**Figure 3:**
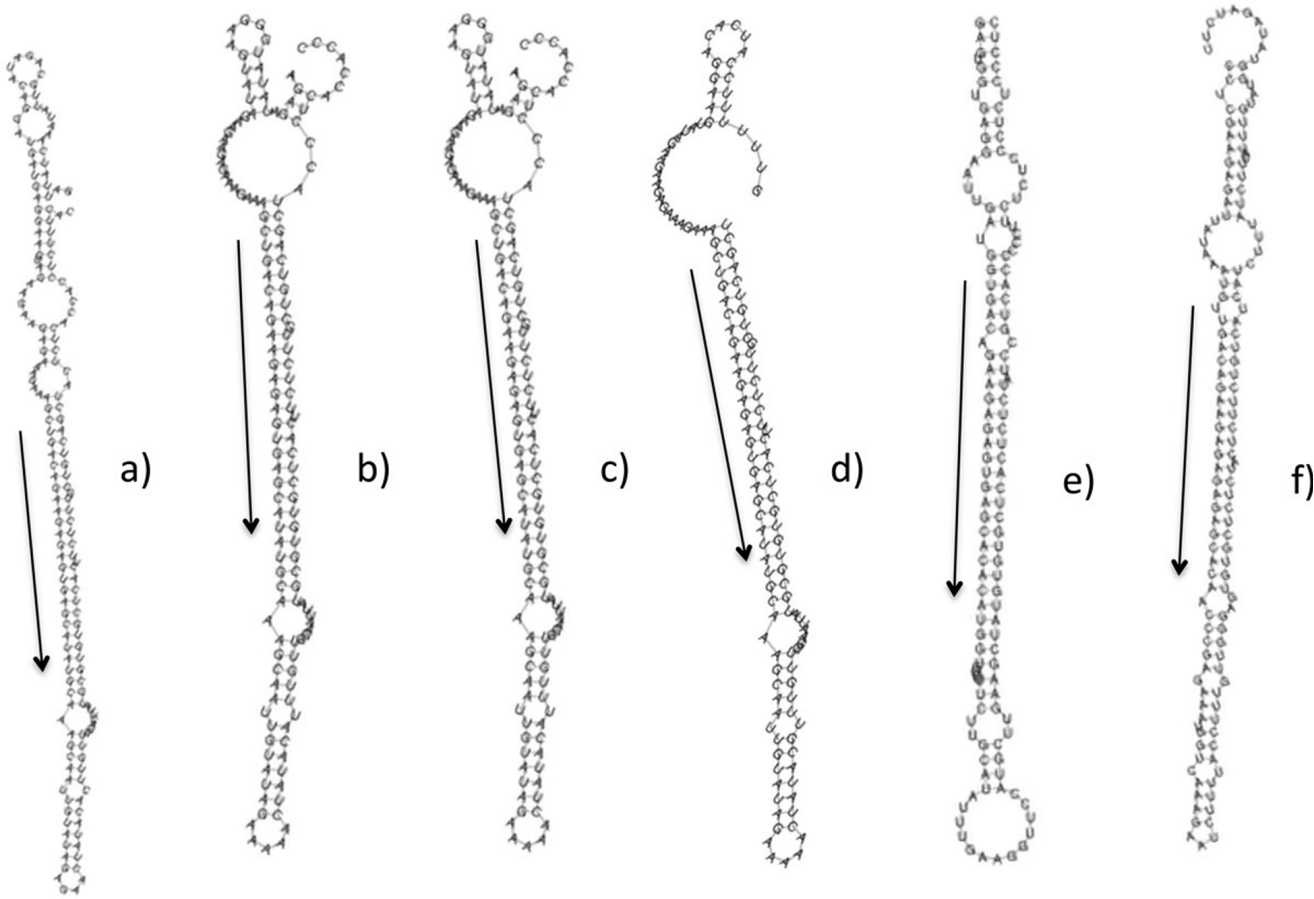
**Similarity of miRNA secondary structures**. Stem-loop of structures of miR156 in Cardueae, acc. GE609552, EL372706, EL373856, EL385094 with mfe -66, -59.8, -58.8, 57.4 kcal/mol respectively (a-d); stem-loop structures of miR156 from Arabidopsis and arachis, (Ath-miR156f and Ahy-miR156c) with mfe -64.12 to -56.6 kcal/mol (e-f).

**Figure 4:**
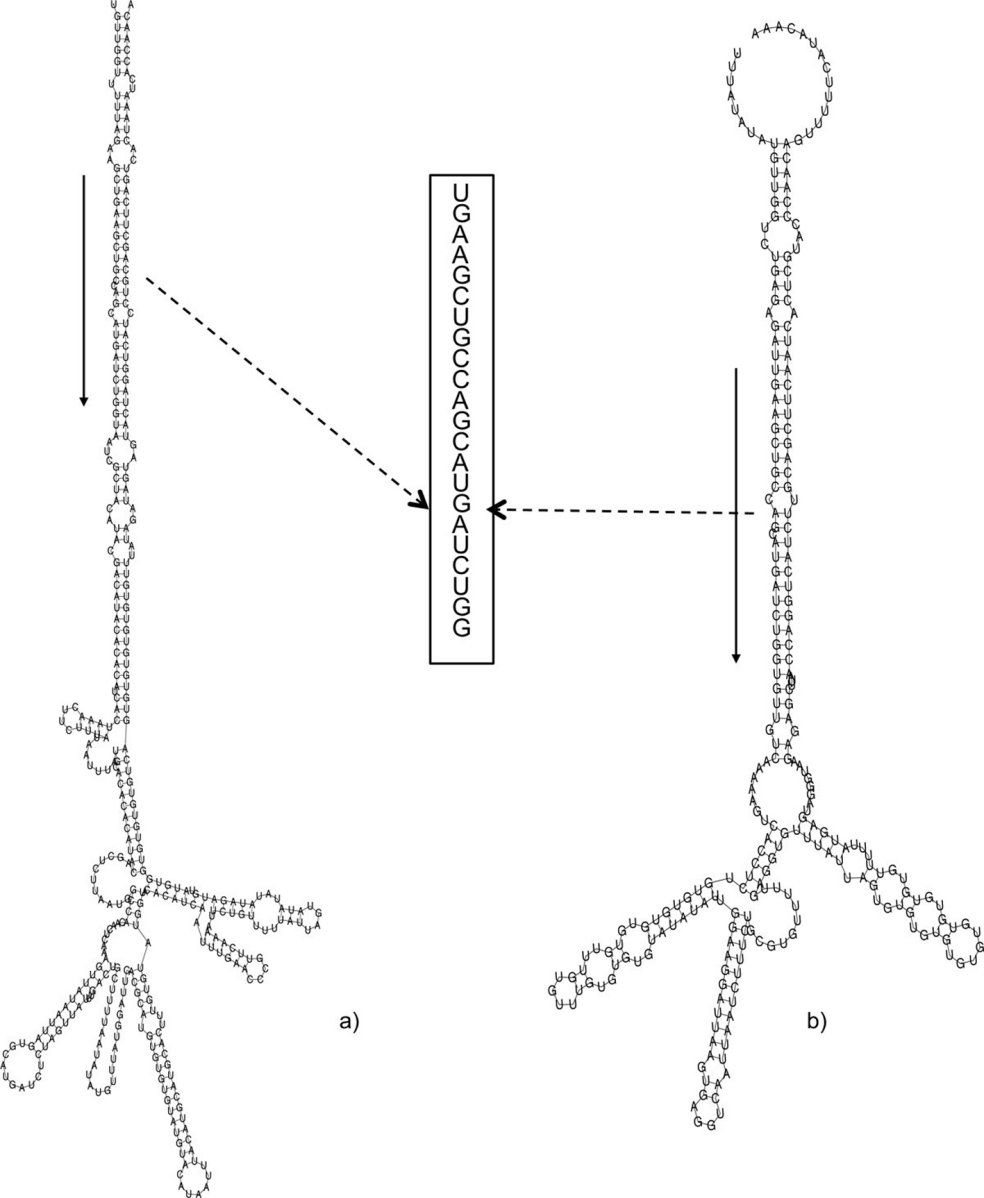
**Location and secondary structures of Ath-miR167d and its homologous in artichoke**. Secondary structure of the Ath-miR167d primary transcript (mfe -129.70 kcal/mol) (a). Predicted secondary structure of *Cynara cardunculus *primary transcript (mfe -79.00 kcal/mol), corresponding to EST GE597437 (b). Arrows indicate the conserved mature miRNA, highlighted in the box.

Einverted program from EMBOSS package [29], was used to find stem loop regions on the complete EST datasets of *C. cardunculus *and *C*. *tinctorius*, containing 36323 and 42011 entries respectively (release 177.0, April 2010, available at NCBI). ESTs with inverted repeats were analysed to find the occurrence of putative mature miRNAs, and their complementary units (miRNA*) sequences using 953 mature miRNA related to 8 dicotyledon and 2 monocotyledon (see methods). The ESTs with two match (miRNA mature and miRNA*) were considered only if the sequence length between the matches is equivalent to the orthologous pre-miRNAs, stored in miRBase [30]. These ESTs were inferred as good candidates for miRNA precursors. The screening followed by analyses of the secondary structure [31] of the putative pre-miRNAs thus allowed identification of 17 miRNA candidates within the two considered species. In particular, using these criteria, three ESTs [EMBL:GE587550, EMBL:GE610628, EMBL:GE605691] coding for the homologous to miR398 were retrieved from the *C. cardunculus *dataset. These entries showed a 350 bp homologous alignment region with identity > 95% and a less conserved, 3' region. The miRNA* strand and mature miRNA of the ath-miR398 homolog were found in the conserved region, in reverse and complementary orientation (Figure 2). When analysed by RNAfold, these regions showed a secondary structure comparable to the *Arabidopsis *miR398a/b/c (minimum free energy, mfe -55.9 Kcal/mol) precursor (additional file 1). After a BLAST [32] search these sequences did not exhibit any significant similarity with known proteins from the Uniprot database (db) pointing out that they might not code for proteins, and suggesting that they could code for a putative miR398 like or homologue in *C. cardunculus*. In the following EST EMBL:GE609552, EMBL:EL372706, EMBL:EL373856, EMBL:EL385094 using the same procedure we identified orthologous for miR156, (Figure 3 and additional file 1). Furthermore, were identified miR167d in EMBL:GE597437 (Figure 4), miR408, miR390, miR834, miR2919, miR4387c, miR396 families corresponding to EMBL:GE605886, EMBL:GE603045, EMBL:GE610451, EMBL:EL394166, EMBL:EL405672, EMBL:GE592223 and EMBL:GE577632, EMBL:EL409447. In addition we used sequences from NGS study [33] find out that sequence, EMBL:EL395532 represents the precursor of cat-m0011.

BLASTx analysis showed at least three miRNAs within coding regions, (E-values range: 1e-26 to 8e-127), corresponding to miR168a and miR834 in *C*. *tinctorius*, and to miR390 and miR834 in *Cynara*. The latter was detected within EMBL:GE610451, that is homologous to the BURP domain-containing proteins (E-value: 6e-24), identified in many plants, putatively involved in a variety of vegetative and reproductive developmental functions, as well as in responses to environmental stresses [34]. MiR390, identified in EMBL:GE603045, showed a high similarity level with chaperonin family Tcp-1/cpn60 [35], (E-value: 8e-127). All sequences of precursors of the above mentioned pre-miRNAs, fold into a perfect hairpin structure with low free energy levels (additional file 1).

### Experimental identification of mature miRNAs

An experimental assay was developed for *in-vitro *isolation of mature miRNAs from *C*. *cardunculus*. Primers were designed for 10 miRNAs, based on the alignment of available *A. thaliana *mature miRNAs and EST sequences of *C. cardunculus *and *C. tinctorius*, considering variable nucleotides at divergent sites (Table 1). Assays were performed on total RNA from artichoke leaves. The first strand (RT) was synthesized using an oligo-dT primer carrying an adaptor. A 3' primer complementary to the adaptor and a 5' primer corresponding to the miRNAs, annealing to their reverse transcribed target, were used for polymerase chain reaction (PCR). In six reactions out of ten, miRNA-RT-PCR produced the predicted 70 base pair fragments, including the adaptor sequences and the stabilisation tails. Cloning and sequencing the PCR products confirmed the identity of four amplified artichoke miRNAs, corresponding to miR171b.c (sequence: TTGAGCCGTACCAATATCACG), miR833-5p (sequence: TGTTTGTTGTACTCGGTCTAGT), miR472 (sequence: TTTTCCTACTCCGCCCATACC), and miR390b (sequence: AAGCTCAGGAGGGATAGCACC). The latter sequence matched the reported miRNA of *Glycine max *that differs from that of *A. thaliana *in the 19^th ^nucleotide, in which there is an A instead of G (underlined in the sequence above). The two miRNAs not represented in the sequenced clones, together with the not amplified miRNAs, suggested either a lack of expression in the artichoke leaves tested, or a very low abundance preventing isolation or misidentification. Three sequenced miRNAs (miR171b.c, miR390b and miR472) appeared highly conserved in plants, due to their occurrence in several species. However, this is the first retrieval within the *Asteraceae *family of miR833-5p, thus far considered specific for *Arabidopsis *only. In spite of the PCR reaction sensitivity, this method allowed the validation of a first set of miRNAs from artichoke leaves. To increase their number, a further step is needed to enrich the fractions of small RNAs. A wider range of plant developmental stages is also required to allow the identification of further miRNAs, involved in other differential regulatory pathways.

**Table 1:** List of primer sequences for RT-PCR analysis for cloning and sequencing microRNAs from *Cynara cardunculus *leaves

Related miRNA	Primer Name	Primer Sequence
ath-miR833-5p	mir833	cgccgctgtttgttgtactcggtctagn
ath-miR157b	mir157	cgccgcttgacagaagatagagnncac
ath-miR472	mir472	cgccgcytttycctactccyccnatncc
ath-miR390b	mir390	cgccgcaagctcaggagggatagcnnc
ath-miR171b.c	mir171	cgccgcttganccntnccaanatcacg
ath-miR171a	mir171a	cgccgctgattgagccgcgccaatnnn
ath-miR832-5p	mir832	cgccgctgctgggatcgggaatcnann
ath-miR169d	mir169	cgccgctgagccaaggatgnnttgcnn
ath-miR397b	mir397	cgccgctcatngagtgnancgttgatn
ath-miR164a	mir164	cgccgctngagaagcagggnacntgca
	Poly(T) adapter	gcgagcacagaattaatacgactcactatagg(t)12vn §
	Poly(T) adapter reverse*	gcgagcacagaattaatacgact

§ degenerated bases are indicated by the following one-letter code: C+T = Y; A+C+G = V; A+C+G+T = N.

Motif cgccgc corresponds to stabilisation tail.

* Common reverse primers for all miRNAs

Identification of homologous regions between *C. cardunculus *and *C. tinctorius*, and the conservation of identical putative targets in both analysed sequences, provided a guide for specific primer design to further assess *C. cardunculus *miRNAs *in vivo*. This approach allowed the experimental validation of 4 out of 10 selected miRNAs, using as template RNAs from young *C. cardunculus *leaves. However, to be able to capture all expressed miRNAs and considering that only young leaves were assayed, it appeared necessary to test various phenological stages or plant developmental phases, in order to get a more complete scenario of the miRNA expression patterns.

However, this study revealed that some sequences considered specific only for *Arabidopsis *are also present in *Carduoideae *(i.e. miR833-5p). All analyses carried out in this study highlighted and confirmed the broad conservation and homology of plant mature miRNAs. Results herein shown strengthen the hypothesis that miRNAs play a fundamental role at the cellular level, and that they are nearly always conserved in related organisms [36]. In particular, their conservation suggests a feedback mechanism of genome stabilization and protection, through selective pressures active at the gene expression level. Mutations in miRNA sequences have in fact a very low probability of being mirrored by complementary changes occurring at their target loci or vice versa, thus setting in place a simple but efficient mechanism of control [37]. However, as for orphan genes, during evolution some structures persist even if their function was lost. Since *A. thaliana *and *Carduoideae *do not share the same evolutionary line, caution is needed when inferring the functionality of the miRNA-target links observed.

### Target prediction

RNAhybrid results showed that 7145 and 8730 ESTs in *C*. *tinctorius *and *C. cardunculus*, respectively, have at least one predicted miRNA target (additional files 2, 3). The selection parameters used in the analysis (see methods) were: maximum one loop, one mismatch, and overhangs not longer than 2 nucleotides and mfe cut off value > 70% of the perfect match obtained by RNAHybrid, and calculated for each miRNA mature sequence [38] (additional file 4). Data also showed a pool of 1443 ESTs, 990 for *C. scolymus *and 453 for *C. tinctorius*, in which repeated (identical or different) targets occur in the same EST. The prediction of multiple targets within the same EST in several positions, suggests the possibility of evolutive mechanisms related to co-regulation or amplification. Data indicate that, in *Asteraceae*, several types of miRNAs could be involved in this mechanism, in a way similar to the multiple and different targets on the same mRNAs proposed in *Arabidopsis*, i.e. miR400 and miR161 that target Tair:At1g06580, Tair:At1g62720 and Tair:At1g62670 mRNAs [39].

### Blast results and COS identification

The comparative approach herein followed allowed the identification of conserved homologous/orthologous sequences (COS regions) in EST datasets of *C. tinctorius *and *C. cardunculus*, pointing out the unique features (shared sequences), present in this phylogenetic group. A systematic search for aligned regions between *C. tinctorius *and *C. cardunculus *ESTs, through BLAST analyses, resulted in the identification of 233891 homologous sequences. We inferred that about 75% of *C. tinctorius *and *C. cardunculus *ESTs shared at least one homologous region (E-value < 10^-4^). The aligned regions longer than 400 bp (E-value < 10^-10 ^and identity > 75%), were considered as significant (additional file 5). They covered about 50% of the complete EST dataset (16921 sequences for *C. cardunculus *and 19582 for *C. tinctorius*) and were considered to represent sequences that code for proteins with similar functions in these two species.

About 8000 *C. cardunculus *and 9900 *C. tinctorius *EST sequences did not show a common region, probably due to incomplete transcriptome sequencing data.

### Conserved target in COS region

Analyses carried out in COS regions resulted in the identification of miRNA targets, conserved in *C. cardunculus *and *C. tinctorius*, obtained after aligning of 960 and 890 ESTs of *Cynara *and *Carthamus *respectively. Following a statistical evaluation of the predicted targets, a pool of 79 (additional file 6) different mature miRNA targets with a signal to noise ratio > 2 and specificity > 0.85 was identified.

The complete EST sequences analyses, including target and relative functions, are reported in additional file 7. ESTs EMBL:GE592822 and EMBL:EL403073 (*C*. *scolymus *and *C. tinctorius*, respectively) showed a 600 bp homologous region in which a miR157a/b/c target was present. Members of this family are ubiquitous within plants, as they were also found in *Fabaceae*, *Vitaceae*, *Solanaceae *and now in *Cardueae *(this study). BLASTx analysis showed a significant similarity of these accessions to *Arabidopsis *cyclophillin [Refseq:NP_200679], an important protein involved in variety of cellular processes in plants, including protein trafficking and maturation, receptor signalling and complex stabilization [40,41]. A target for miR397a was predicted on a COS region [EMBL:GE583552 and EMBL:EL386866 for *C. cardunculus *and *C. tinctorius*, respectively], similar to laccase [Refseq:NP_187533], a multi-copper containing glycoprotein, member of a multigene family present in plants as well as other organisms [42]. Furthermore, miR398 was predicted in COS region with specificity > 0.85; comparison analysis of these ESTs [EMBL:GE585699, EMBL:GE592094, EMBL:GE589103, EMBL:GE602849] against the *Arabidopsis *proteome showed significant similarity to copper/zinc superoxide dismutase (Cu,Zn-SOD) [Refseq:NP_56566, Refseq:NP_001077494]. In *Arabidopsis*, down-regulation of Cu,Zn-SOD expression involves miR398, that directs the degradation of its mRNA at Cu^++ ^low concentrations [43]. Three additional miRNA families, miR397 (found in this study), miR408 and miR857 were predicted in *Arabidopsis *and other plants to target transcripts for the Cu^++ ^protein plastocyanin and members of the laccase family, as shown by their accumulation in response to low Cu^++ ^concentrations, demonstrating a microRNA-mediated down regulation [44]. Furthermore, the four miRNAs above mentioned are known to perform important functions in Cu^++ ^homeostasis within the plant cells [45]. Moreover, other BLASTx analyses against the *Arabidopsis *proteome showed similarity of *C. cardunculus *with a nucleotide binding protein and to another protein with unknown function (additional file 7).

In this study, potential targets for miR390 were found within 12 ESTs of *C. cardunculus*, whereas only 6 were conserved in *C. tinctorius *COS regions. In *Arabidopsis*, mature ath-miR390a is obtained by two precursors [miRBase:MI0001000 and miRBase:MI0001001]. The mature sequence of ath-miR390a is ubiquitous in plants, since it is shared with other 24 species of Eudicotyledons, Monocotyledons as well as *Physcomitrella patens *(Embryophyta). A homologous sequence is also known in Coniferophyta (*Pinus taeda*) with a G→U substitution in position 11 [46]. Ath-miR390 drives the cleavage of the non-protein-coding primary transcripts of trans-acting (ta) genes directing the formation of small-interfering ta-siRNAs. These miRNAs are involved in the cleavage of AGO proteins, with RNaseH-like activity cleaving ta-siRNAs single-stranded RNA transcripts in the region complementary to small RNAs [47]. Two miR390 target sites (5' and 3' ta-siRNA sites) were shown to be necessary in *Arabidopsis *for ta-siRNAs 3 precursor RNA cleavage, dependent on a specific interaction between AGO7 and miR390, suggesting a similar putative role of miR390 in the *Cardueae *(additional file 7).

Within the *C. cardunculus *transcriptome, this pool of miRNAs conserved in COS regions was considered trustable for subsequent *in vivo *validation, since their identification was experimentally more probable than the vertical prevision carried out by RNAhybrid in each of the two datasets, separately.

Eight *C. cardunculus *EST sequences (EMBL:GE577936, EMBL:GE578910, EMBL:GE582576, EMBL:GE586807, EMBL:GE606316, EMBL:GE607689, EMBL:GE608348, EMBL:GE610548) were found as possible targets of ath-miR400 (signal to noise ratio: 26, specificity: 0.96). So far, ath-miR400 was considered specific in *Arabidopsis *[miRBase:MIMAT0001001 and TAIR:AT1G32582]. BLASTx analysis of these ESTs showed a significant similarity with an *Arabidopsis *26 proteasome subunit-like protein [Uniprot:Q8RWF0]. In *Arabidopsis*, miR400 is predicted to target more than 10 genes of the pentatricopeptide repeat (PPR) protein family, characterized by tandem repeats of a degenerate 35 amino acid motif. Some proteins play a role in post-transcriptional processes within organelles, current evidence suggesting that PPR proteins bind RNA as well as other proteins [38,48,49].

## Conclusions

The confirmed role played by miRNAs in both physiological and pathological processes make them an interesting object of study [45]. Herein we followed a comparative transcriptome *in silico *approach that let us mining for putative miRNAs and their possible targets in globe artichoke and safflower. Using this procedure we identified 17 miRNAs in both species and after statistical analysis 79 conserved targets, found on the COS regions and belonging to 60 miRNA families, with a signal to noise ratio > 2, and ≥ 0.85 specificity, allowing for their *in vivo *functional analyses.

The identification of 17 ESTs codifying for miRNA precursors in Asteraceae family, showed that the rate of precursors retrieve is estimated at 0.021%, alike to the real rate found in Arabidopsis (see material and methods), highlighting the validity of the developed method; moreover catm0011, a novel miRNA predicted from sufflower [33], let us to retrieve a EST that codified the entire precursor (additional file 1). The complementarity between miRNAs, targets and sequence conservation in Cynara and Carthamus helped us predict regulated genes, as well. The bioinformatic approach identified ESTs whose sequences were maintained in both species as most probable targets. The occurrence and mining of COS regions from both species validated the analysed targets, suggesting that many true positives were also possibly missed. In fact, any real target identified in one species was ignored if the counterpart sequence was not found in the other species, a problem occurring when the ESTs in public databases do not represent the whole transcriptome. The presence of multiple targets within a gene agrees with the concept of cooperatively acting miRNAs. Finally, a possible outcome of our approach would be the development of bioinformatic tools detecting multi-species COS regions using publicly available ESTs, altogether with an evaluation of their level of conservation. In fact, recent data concerning miRNAs identified in safflower [33] confirmed the validity of the *in-silico *approach herein applied, since 52% of the miRNA groups identified in this study were also present in the deep sequencing data produced from different organs of this plant.

## Methods

### *In silico *analysis

#### Identification of putative conserved miRNAs and their precursors

A computational approach to identify potential miRNA precursors was carried out in the EST db of *C. cardunculus*. Einverted program of the EMBOSS package [29] was employed to search in the *Cynara *dataset the inverted repeats longer than 30 bp. The results acquired were included in the database and used in the next step by RNAHybrid analysis, searching the putative mature miRNAs and miRNA* strand. The ESTs derived after the analysis were stored in a dedicated board in the database and used for the extraction of 200 bp region around the putative miRNA targets present in the inverted repeat. The 200 bp regions further used in the RNAfold analyses and the mfe-estimate and hairpin structure prediction have been compared with orthologous precursors from the following species *Arabidopsis*, *Arachis hypogaea*, *Solanum lycopersicum*, *Medicago truncatula*, *Populus trichocarpa*, *Gossypium hirsutum*, *Zea mays*, *Oryza sativa*, *Vitis vinifera *and *Citrus*. Furthermore we tested method accuracy on Arabidopsis EST dataset, by a BLAST analysis, using as targets 1.065.779 ESTs, and as query the Arabidopsis precursors. The analyses were conducted under stringent conditions, that consider a 90% identity, and pre-micro sequence alignment coverage ≥ 70%, in order to limit the false positive arose from the possible targets. The analysis identified 221 EST sequences, which represent a rate of 0.020%. On the same Arabidopsis EST dataset, our bioinformatic approach recognised 196 out of 221, corresponding to 0.018% rate of EST sequences present. (See also additional files 8 and 9).

#### Identification of conserved homologous/orthologous EST regions

The BLAST program was used to identify homologous regions within the ESTs of the species under study. In the BLASTn (e-value e10^-4^) analysis we considered *C. cardunculus *ESTs as query and *C. tinctorius *ESTs as target. The results obtained were stored in the relational database and used by MySQL for the extraction of COS regions longer than 400 bp (we considered 400 pb a significant value of an aligned region comparable to mean EST length in these two datasets), e-value < 10^-10 ^and identity higher than 75%.

#### Target prediction analysis

The RNAhybrid program was used to identify possible binding sites present in the ESTs, following specific base pairing rules. RNAhybrid finds the mfe hybridisation of two RNAs fragments with different lengths, i.e. long (ESTs) and short (mature miRNA sequences), respectively [27,28].

The *Arabidopsis *mature miRNA sequences were extracted from miRNA.dat file available at miRbase release 17, 2011 [30] including all published miRNA data in EMBL format, by means of an in house developed Perl and MySQL script. The miRNA.dat file contains 1581 miRNA hairpins precursor for the species analysed, from which we obtained 1803 mature miRNA sequences (in some cases the precursor give rises to more than one mature sequences). After comparative analysis of the mature miRNA, considering a perfect match, 953 non redundant mature miRNAs were identified. The two EST datasets were used as target sequences in forward and reverse mode, considering *Arabidopsis *mature miRNA sequences as query. The parameters used in the analysis were: maximum loop size: 1 nucleotide; maximum mismatch size: 1 nucleotide, overhangs: 2 nucleotides, the mfe considered for each target was higher than 70% of the minimum value assessable on perfect match between the mature miRNA and its target.

#### Statistical target evaluation on COS regions

In order to evaluate the confidence level of the target prediction on the COS regions, for each target the number of occurrences found was confronted with those expected in random samples. To assess the signal-to-noise ratio and specificity level, two approaches were employed: in the first one, for each mature miRNA sequence used in the analysis, 10 randomized sequences were produced, with the same di-nucleotide composition of the source, by means of shuffleseq program [29]. In the second approach for the homologous sequences (960 and 890 ESTs in *C. cardunculus *and *C. tinctorius *respectively), in which an identical target was present in the COS region, 10 randomized sequences were produced with the same nucleotide compositions of the sequence used as source. The two datasets of the random sequences were used in the RNAhybrid analysis. The occurrence (false positive) was achieved by two approaches, first the random mature miRNAs were employed as query sequences and the real EST datasets as target. The latter method employed the random EST dataset as target and the real mature miRNA sequences as the query. The average of predicted targets occurrence (false positive) obtained by these two approaches was compared with real miRNAs targets, obtained by previous analyses, that contained either the true positive (TP) and the false positive (FP) matches. For each miRNA, the occurrence obtained in the shuffle analysis was considered as false positive. Therefore, the specificity (S) was defined as follows:

S=TP/(TP+FP)

And the signal to noise ratio (SNR) as:

SNR=(TP+FP)/FP

The specificity as function of the signal-to-noise ratio is:

S=1-(1/SNR)

Where *(TP + FP) *is the number of all predicted miRNA:target relationships on authentic miRNAs versus authentic mRNAs, and FP is obtained from the randomized data.

### Experimental assays

#### Plant growth

Seedlings of globe artichoke (*C. cardunculus *var. *scolymus *L.) F1 hybrid (Harmony green variety, Nunhems, NL), were grown in a glasshouse at 22 ± 2°C, with a photoperiod of 16/8 h (light/dark). All plants were subjected to the same general care, including fertilization and watering regimes.

#### RNA extraction

Total RNA was extracted from leaf (cotyledon and true leaves) of 3-weeks old globe artichoke plants, using Trizol reagent (Invitrogen, UK) followed by RNase-free DNase digestion (RNase-free DNase set and RNeasy Mini Kit, Qiagen GmbH, Germany). Samples concentration was determined by a spectrophotometer (NanoDrop^©^, Thermo Scientific, USA). Polyadenylation was performed as follows: 2 μg of total RNA were added to 25 μl of a reaction mixture in presence of 2.5 mM MnCl_2_, 80 μM ATP, 1× reaction buffer and 1 U of *Escherichia coli *Poly(A) Polymerase from Ambion's Poly(A) Tailing kit (Applied Biosystems, US). Subsequently, the reaction mixture was incubated for 15 min at 37°C to add a short adenine tail to the non-poly(A) low molecular weight RNA molecules [50]. An aliquot of 4 μl (320 ng) of the polyadenylated RNA was reverse transcribed with a Poly(T) adapter in presence of High Capacity Reverse Transcription Reagents (Applied Biosystems, USA), performed at 25°C for 10 min followed by 50 min at 42°C, and finally 1 min at 85°C.

Amplification of miRNAs was carried out from 1 μl of a 1:10 dilution of the reverse transcribed reaction with a common reverse primer, Poly(T) adapter reverse, homologous to 23 nt in the Poly(T) adapter sequence, while the forward primers were chosen according to homologous sequences from miRNAs selected through the *in silico *analysis. Ten miRNAs were selected representing an assortment of *Arabidopsis *miRNA families that showed conservation in globe artichoke and safflower. EST databases with a specificity value higher than 65%. The primers design considered introduction of degenerated nucleotides to match multiple nucleotides present in the same positions in the three sequences considered. A 6-nt sequence was also included at the 5'- terminus to stabilize the amplification reaction [51]. The reaction was carried out in a volume of 25 μl containing 200 nM primers (see table 1) dNTP, buffer. Cycling profile were 95°C for 2 min, followed by 40 cycles of 10 s at 95°C, 20 s at 55°C and 20 s at 72°C. Amplified products were cloned into pGEM-T vector and used to transform competent *Escherichia coli *DH5α cells. Plasmid containing inserts were used for DNA automated sequencing services (Macrogen, South Korea).

## List of abbreviations used

miRNA: microRNA; RISC: RNA-induced silencing complex; AGO: ARGONAUTE; dsRBP: dsRNA binding protein; ESTs: Expressed sequenced tags; COS: Conserved homologous/orthologous sequences; mfe: minimum free energy; NGS: next generation sequencing; PPR: pentatricopeptide repeat; Cu, Zn-SOD: copper/zinc superoxide dismutase.

## Competing interests

The authors declare that they have no competing interests.

## Authors' contributions

DC and MMFS conceived and designed the study. DC carried out the bioinformatic analyses, performed the statistics and helped to draft the manuscript. MMFS performed the experimental assays and drafted the manuscript. DP and GS participated in the design of the study, the discussion of results and helped to draft the manuscript. All authors read and approved the final manuscript.

## Supplementary Material

Additional file 1 **predicted pre-miRNAs folding in Compositae**. The table contain the information about all pre-miRNAs present in *Carthamus tinctorius *and *Cynara cardunculus *EST datasets. The table lists Embl/Genbank accession numbers, the fold image of the Cynara/Carthamus precursor and the orthologous extracted from miRBase database. In the last two columns we have reported the sub-sequence necessary to obtain the secondary structures. Click here for file

Additional file 2 **RNAhybrid results in *Carthamus tinctorius *ESTs**. The table lists the results of target prediction analysis carried out by RNAhybrid within the ESTs of *Carthamus tinctorius*. The parameter used in the analysis were: maximum one loop, one mismatch, and overhangs not longer than 2 nucleotides and mfe cut off value > 70% of the perfect match obtained by RNAHybrid. Click here for file

Additional file 3 **RNAhybrid results in *Cynara cardunculus *ESTs**. The table displays the RNAhybrid analyses of the target prediction within the ESTs of *Cynara cardunculus*. The parameter used in the analysis were maximum one loop, one mismatch, and overhangs not longer than 2 nucleotides and mfe cut off value > 70% of the perfect match obtained by RNAHybrid. Click here for file

Additional file 4 **Minimum free energy calculation for each miRNA target, by RNAhybrid**. In the first and the second columns are reported the mirBase identifier and accessions numbers. In these two columns, the miRBase ID/accession have been listed by comma, for the species with identical mature miRNA sequence. For each mature miRNA the mfe and mfecut-off, used in the analysis, is indicated. Click here for file

Additional file 5 **Identified COS regions in *Carthamus tinctorius *and *Cynara cardunculus***. The table displays the COS regions shared by *Carthamus tinctorius *and *Cynara cardunculus*. Only the alignment of 400 bp or longer present in both species was considered. The position and the length of the fragment shared are recorded within each EST, as well as the values obtained in the BLAST analysis. Click here for file

Additional file 6 **Significant identified miRNA targets**. The table contains the significant targets (specificity > 0.8) identified by this pipeline. For each target has been indicated the occurrence find in the ESTs, the average of the occurrence obtained considering two shuffled sequence datasets, the signal to noise ratio and the specificity. Click here for file

Additional file 7 **Significant target functions**. In the table are showed the statistical significant targets and the putative function obtained by comparison of the *Cynara *ESTs (representing one COS) against the *Arabidopsis *proteome. Click here for file

Additional file 8 **Arabidopsis Test 1**. The table depicts 221 EST sequences as real pre-miRNA retrieved after BLAST analyses in Arabidopsis EST dataset. The analyses were conducted considering identity > 90%, and pre-micro sequence alignment coverage ≥ 70%, in order to limit the false positive originated from the possible targets. Click here for file

Additional file 9 **Arabidopsis Test 2**. In the table are listed the pre-miRNA obtained on the same Arabidopsis EST dataset used for the real pre-miRNA identification (see Table S8), by the bioinformatic approach described in the manuscript. We recognised 196 out of 221, corresponding to 88% of the real precursor present in the data. Click here for file
